# Cost of Delivering 12-Dose Isoniazid and Rifapentine Versus 6 Months of Isoniazid for Tuberculosis Infection in a High-Burden Setting

**DOI:** 10.1093/cid/ciaa1835

**Published:** 2020-12-07

**Authors:** Courtney M Yuen, Arman Majidulla, Maria Jaswal, Nauman Safdar, Amyn A Malik, Aamir J Khan, Mercedes C Becerra, Salmaan Keshavjee, Chunling Lu, Hamidah Hussain

**Affiliations:** 1 Division of Global Health Equity, Brigham and Women’s Hospital, Boston, Massachusetts, USA; 2 Department of Global Health and Social Medicine, Harvard Medical School, Boston, Massachusetts, USA; 3 Harvard Medical School Center for Global Health Delivery, Boston, Massachusetts, USA; 4 Interactive Research and Development, Karachi, Pakistan; 5 Global Health Directorate, Indus Health Network, Karachi, Pakistan; 6 Interactive Research and Development (IRD) Global, Singapore

**Keywords:** latent tuberculosis, chemoprevention, costs and cost analysis, rifapentine, Pakistan

## Abstract

**Background:**

Successful delivery and completion of tuberculosis preventive treatment are necessary for tuberculosis elimination. Shorter preventive treatment regimens currently have higher medication costs, but patients spend less time in care and are more likely to complete treatment. It is unknown how economic costs of successful delivery differ between longer and shorter regimens in high-tuberculosis-burden settings.

**Methods:**

We developed survey instruments to collect costs from program and patient sources, considering costs incurred from when household contacts first entered the health system. We compared the cost per completed course of preventive treatment with either 6 months of daily isoniazid (6H) or 3 months of weekly isoniazid and rifapentine (3HP), delivered by the Indus Health Network tuberculosis program in Karachi, Pakistan, between October 2016 and February 2018.

**Results:**

During this period, 459 individuals initiated 6H and 643 initiated 3HP; 39% and 61% completed treatment, respectively. Considering costs to both the program and care recipients, the cost per completed course was 394 US dollars (USD) for 6H and 333 USD for 3HP. Using a new 2020 price for rifapentine reduced the cost per completed course of 3HP to 290 USD. Under varying assumptions about drug prices and costs incurred by care recipients, the cost per completed course was lower for 3HP in all scenarios, and the largest cost drivers were the salaries of clinical staff.

**Conclusions:**

In a high-burden setting, the cost of successful delivery of 3HP was lower than that of 6H, driven by higher completion.

## Teaser

In a tuberculosis-preventive treatment program in Karachi, Pakistan, the cost per completed course was lower for 12-dose isoniazid and rifapentine than for 6 months of daily isoniazid.

Treatment of tuberculosis infection is a critical component of the strategy to eliminate tuberculosis [1, 2]. Household members of patients with tuberculosis are at high risk for tuberculosis infection, and 5–10% will progress to disease within 2 years [3]. In low- and middle-income countries, 3% of household contacts of people with tuberculosis have been found to have tuberculosis disease themselves, and 45% to have latent infection [4]. Giving tuberculosis preventive treatment to household contacts of patients with tuberculosis is a pillar of tuberculosis elimination [1] and appears in most national tuberculosis guidelines [5]. However, there is an implementation gap in delivering preventive treatment in settings with high tuberculosis burdens, and few contacts make it to end of the tuberculosis preventive treatment cascade [6, 7].

One major challenge in delivering tuberculosis preventive treatment is ensuring treatment completion. Studies from varied settings have found that less than half of household members who start preventive treatment complete it [6, 7]. In high-income settings, programs have shifted away from using longer regimens of 6–9 months of daily isoniazid in favor of shorter 3–4-month rifamycin-based regimens and have found that patients receiving shorter regimens are more likely to complete treatment [8–10]. Currently, the medications used for the shorter regimens have higher prices than isoniazid. However, analyses from Canada and Australia have shown that most of the cost of delivering the preventive treatment comes from the cost of clinical visits, and using shorter regimens reduces overall costs by reducing the number of clinical visits required [11, 12].

In the low- and middle-income countries where the global tuberculosis burden is concentrated, 6 months of isoniazid (6H) has long been the only preventive treatment regimen available. A 2018 World Health Organization (WHO) policy update endorsing the option of shortened regimens superseded previous WHO policy recommendations that those regimens should be considered only by high-income countries [13]. Despite this change, the higher cost of medications in the shortened regimens has caused policymakers and stakeholders to hesitate in adopting them. For example, the 2020 Global Drug Facility (GDF) price for 1 course of a 12-dose weekly isoniazid and rifapentine regimen (3HP) is 5–15 times the price for 1 course of 6H [14]. In high-income countries, the cost savings from the reduced length of time that patients are in care offsets the increased cost of medications [11, 12]. No studies have assessed whether this holds true in countries with high tuberculosis burdens, where some healthcare delivery costs are lower.

In 2017, the Indus Health Network’s (IHN) tuberculosis program in Karachi, Pakistan, became one of the first programs in a high-tuberculosis-burden country to use 3HP programmatically. To compare the costs of delivering 3HP versus 6H in a high-burden setting, we conducted a cost analysis of this program, assessing economic costs per completed course for the 2 regimens.

## METHODS

### Study Setting

Pakistan is a lower-middle-income country with a WHO-estimated tuberculosis incidence of 265 per 100 000 population [15]. The IHN provides free services to all patients. Since 2007, it has partnered with the National Tuberculosis Program to provide tuberculosis services in its tuberculosis clinics.

This analysis focuses on the cohort of tuberculosis household contacts who initiated 6H or 3HP preventive treatment during October 2016–February 2018. As part of the IHN tuberculosis program’s contact-management procedures, patients with tuberculosis were counselled to bring their household members for evaluation. Evaluation included chest radiography, GeneXpert MTB/RIF, Sunnyvale, CA, Cepheid (if contacts could produce sputum), and clinical examination. Preventive treatment was initiated once tuberculosis disease was ruled out. For contacts of patients with drug-sensitive tuberculosis, the program switched from using 6H to using 3HP in May 2017, except for children younger than 2 years old who continued to receive 6H. Contacts of patients with drug-resistant tuberculosis also received preventive treatment [16] but were not included in this analysis. There was no routine laboratory monitoring; liver function tests were performed only if clinically indicated.

The initial evaluation to determine eligibility for preventive treatment required 2 visits, with preventive treatment initiated on the second visit. After that, treatment was self-administered. People receiving 6H returned to the clinic once every 2 months and those receiving 3HP returned to the clinic monthly for monitoring visits, with a final visit at the end of treatment. Medications were dispensed at follow-up visits. Treatment completion, defined as finishing 5 or more months of isoniazid for 6H or 11 or more out of 12 doses of 3HP [17], was verified by healthcare workers and documented in the medical record.

### Study Design

We sought to compare the program and patient costs associated with delivering 6H versus 3HP at the IHN tuberculosis program. Henceforth, “patient” refers to a person who received preventive treatment. The outcome of interest was the total cost per completed course of preventive treatment. This outcome is used in cost comparisons of preventive treatment [11, 12] because it measures the cost per successful outcome achieved, not simply the cost of operating a treatment program. We measured costs starting from when a patient first came to the hospital to be evaluated for preventive treatment and ending on the last visit. We assessed costs for patients who initiated preventive treatment during October 2016–February 2018.

We developed survey instruments for collecting cost data at the program and patient levels. The program-level instrument was constructed around a WHO framework, which describes a health system in terms of 6 core components: service delivery, health workforce, health information systems, essential medicines and technology, financing, and leadership and governance [18]. This framework allows analysis of how costs are divided across the 6 components, which is useful for program budgeting and planning. We adapted a previously developed survey instrument [19] to fit the local context. The patient-level instrument captured medical (eg, user fees) and nonmedical costs (eg, travel, food, opportunity cost) to patients for a preventive treatment visit.

We adjusted costs for inflation over the study period to express all costs in 2019 US dollars (USD). Medication-associated costs were directly calculated in USD; all other costs were converted from Pakistani rupees (PKR) (1 USD = 149.1 PKR).

### Cost Data Collection and Estimation

Medication-associated costs included the procurement and shipping costs of isoniazid and rifapentine from GDF during 2016–2018. We assumed that 1 full treatment course (Supplementary Table 1) was used per patient initiating treatment, reflecting the practice of setting aside a full course for each patient at the time of initiation to avoid treatment interruptions in case of stock-outs. We obtained from the National Tuberculosis Program the fee structure for procuring and shipping medications from GDF and used GDF drug prices from the study period. We also conducted a sensitivity analysis using a lower price (15 USD per adult regimen) for rifapentine that became available to 100 countries in 2020 [14].

Non–medication-associated costs were collected from the records of the IHN Finance and Procurement departments. These included staff salaries, diagnostic tests to rule out tuberculosis disease before the initiation of preventive treatment, medical supplies used during clinic visits, computers, phones, and a portion of the clinic utilities. As IHN owns its facilities and no new infrastructure was required, we did not include capital costs. We multiplied monthly costs by 17 months to estimate total nonmedication costs during the study period. We then divided by the number of total preventive treatment patient visits made to obtain the per-visit cost. As nonmedication costs are not sensitive to age group or treatment type, we applied the same per-visit cost estimate to both age groups and regimens.

To estimate patient-level costs per visit, we administered a survey with written informed consent to a convenience sample (N = 100) of people attending preventive treatment visits at an IHN tuberculosis clinic in 2019. The sample included 50 guardians of children receiving 6H, 41 guardians of children receiving 3HP, and 9 adults receiving 3HP. Survey respondents reported their total spending during the visit, the amount of time spent in transit and at the hospital, their average monthly wage, and the number of family members present at the visit. We used the visit time and the respondents’ monthly wages to estimate their lost wages. We estimated lower-bound, upper-bound, and midrange estimates for the opportunity costs to the entire family. The lower-bound estimate assumed lost wages only for the respondent. The upper-bound estimate assumed equal lost wages for all family members, except for the child patient if the respondent was a guardian. The midrange estimate was the average of the high and low estimates. The average cost over the 100 survey respondents was used as the patient-level cost per visit. Because we did not expect patient costs to be affected by the regimen being received, we applied this cost regardless of regimen or age.

Additional details about cost data collection and estimation methods are provided in the Supplementary Material.

### Analysis

For both 3HP and 6H, we multiplied medication-associated costs by the number of patients initiating preventive treatment, as determined from the electronic medical record. We stratified analysis by age group, defining children as those younger than 15 years old. We multiplied the total number of visits made by these patients by the per-visit nonmedication program costs and the per-visit patient costs. We then summed all of these costs and divided by the number of patients who completed treatment to calculate the cost per completed course of preventive treatment.

## RESULTS

During the study period, 459 patients initiated 6H and 643 patients initiated 3HP; for both groups, 53% of patients were children (Table 1). In this cohort, 6H was completed by 39% of patients while 3HP was completed by 61% of patients. Completion was better for 3HP for both children and adults.

**Table 1.** Household Contacts Initiating 6H or 3HP at Indus Health Network Tuberculosis Program During October 2016–February 2018

	Patients Initiating 6H			Patients Initiating 3HP		
	Children	Adults	All	Children^a^	Adults	All
Mean age, years	8	32	19	8	33	20
Number who initiated treatment	242	217	459	338	305	643
Number (%) who completed treatment	102 (42)	71 (33)	173 (39)	209 (62)	177 (58)	386 (61)
Mean number of visits per patient	3.6	3.2	3.4	3.8	3.7	3.8

Abbreviations: 3HP, 3 months of weekly isoniazid and rifapentine; 6H, 6 months of daily isoniazid.

^a^3HP was not used for children <2 years old.

After adjustment to 2019 USD, the isoniazid medication cost per regimen for 6H paid by the program was 3.46 USD for adults and 3.35 USD for children. The cost of isoniazid and rifapentine medications for 3HP was 46.43 USD for adults and 23.46 USD for children. Additional fees for procurement, shipping, insurance, and customs were levied at 12.2% of medication costs. We estimated the total nonmedication program costs for bringing this cohort of contacts into care, evaluating them, and monitoring preventive treatment to be 31.51 USD per visit (Table 2). The major cost driver of nonmedication program costs was clinical staff salaries. No patients had adverse events that required hospitalization, so costs reflect outpatient services.

**Table 2.** Program-Level and Patient-Level Costs Per Visit for Drug-Sensitive Tuberculosis Preventive Treatment During 2016–2018 at Indus Health Network Tuberculosis Program Clinics, in 2019 US Dollars

Type and Category	Components Included	Are These Costs Already Included in Budgets of TB Programs That Do Not Provide Preventive Treatment?	Cost per Visit, USD
Program costs (not including medications)			
** **Health workforce	Program staff salaries, program staff training	No; monitoring preventive treatment will require additional staff time	17.52
** **Supervision and governance	Supervisory staff salaries	Partly; additional effort may be required if preventive treatment is a new activity to supervise	5.63
** **Medical procedures and supplies	Chest X-ray, GeneXpert, and other tests involved in ruling out TB; medical supplies used during visits	Partly; if contact investigations are already being done to diagnose TB disease	5.77
** **Health information system	Data manager salary, computers, phones, data plans	Yes; existing systems can be adapted	2.23
** **Utilities	Water, gas, electricity	Yes; existing clinics can be used	0.37
Total nonmedication program cost per visit			31.51
Patient costs			
Transportation	Transportation to and from hospital		2.08
Food	Food and beverages purchased during trip		0.84
Medications	Patients were not charged for preventive treatment medications, but some reported buying other medications during the trip to the hospital		0.30
Lost wages	Lower-bound: only 1 family member lost wages		3.16
	Midrange: average of lower and upper-bound estimates		7.87
	Upper-bound: all members family members attending visit lost wages, except for child patient		12.57
Total patient cost per visit (lower-bound)			6.38
Total patient cost per visit (midrange)			11.09
Total patient cost per visit (upper-bound)			15.79

Abbreviations: TB, tuberculosis; USD, US dollars.

The midrange estimate of the cost to patients was 11.09 USD per visit (Table 2). If we assumed that only a single member of each family attending the visit was wage-earning (ie, no other family members bore opportunity costs), then the cost per visit dropped to 6.38 USD (lower-bound estimate). If we assumed that all members of the family attending the visit other than child patients were wage-earning, then the cost per visit rose to 15.79 USD (upper-bound estimate).

**Figure 1.**
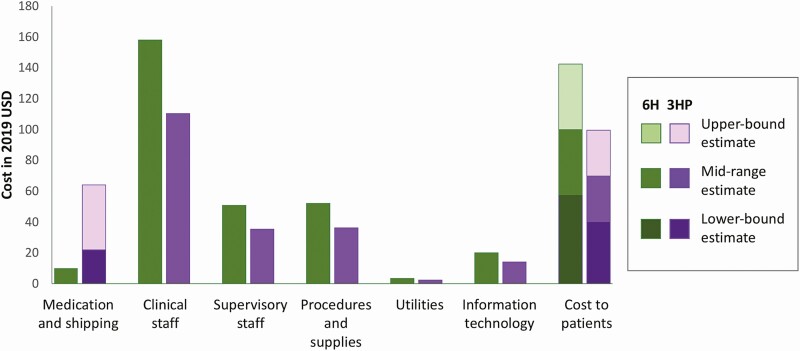
** **Contributors to total cost per course completed of 6H and 3HP. Abbreviations: USD, US dollars; 3HP, 3 months of weekly isoniazid and rifapentine; 6H, 6 months of daily isoniazid.

The midrange estimate for the total cost per completed course of preventive treatment was 394 USD for 6H and 333 USD for 3HP (Table 3). The cost per patient who initiated treatment was 144 USD for 6H and 200 USD for 3HP. When medication costs were calculated using the reduced 2020 GDF price for rifapentine, the midrange cost per course completed was 290 USD for 3HP (Table 4). Under all cost assumptions modeled, the cost per completed course for 3HP was lower than for 6H. The largest cost driver was clinical staff (Figure 1); medication-associated costs comprised 2–21% of total costs (Table 4).

**Table 3.** Calculation of Costs per Completed Course of 6H and 3HP at Indus Health Network Tuberculosis Program Clinics During 2016–2018, in 2019 US Dollars

	6H			3HP		
Cost Category (per Patient or per Visit)	Cost per Patient or Visit, USD	Number of Patients or Visits	Total Cost, USD	Cost per Patient or Visit, USD	Number of Patients or Visits	Total Cost, USD
Adult medications, including shipping (per patient)	3.89	217	843	52.09	305	15 888
Child medications, including shipping (per patient)	3.76	242	909	26.33	338	8898
Health workforce (per visit)	17.52	1560	27 324	17.52	2432	42 598
Supervision and governance cost (per visit)	5.63	1560	8779	5.63	2432	13 687
Medical procedures and supplies (per visit)	5.77	1560	8993	5.77	2432	14 020
Health information systems (per visit)	2.23	1560	3472	2.23	2432	5412
Utilities (per visit)	0.37	1560	580	0.37	2432	904
Patient costs, midrange estimate (per visit)	11.09	1560	17 295	11.09	2432	25 962
Total costs			68 195			128 370
Patients who completed treatment			173			386
Cost per patient initiating treatment			144			200
Cost per course completed			394			333

Abbreviations: USD, US dollars; 3HP, 3 months of weekly isoniazid and rifapentine; 6H, 6 months of daily isoniazid.

**Table 4.** Costs per Course Completed for 6H and 3HP at Indus Health Network Tuberculosis Program Clinics, Under Varying Assumptions of Medication and Patient Costs

	6H		3HP	
Rifapentine Drug Price^a^ and Patient Cost Estimate	Cost per Course Completed, 2019 USD	% of Cost That Is Medications^b^	Cost per Course Completed, 2019 USD	% of Cost That Is Medications^b^
2018 GDF				
** **Lower-bound	352	3	303	21
** **Midrange	394	3	333	19
** **Upper-bound	437	2	362	18
2020 GDF				
** **Lower-bound	352	3	261	8
** **Midrange	394	3	290	8
** **Upper-bound	437	2	320	7

Abbreviations: GDF, Global Drug Facility; USD, US dollars; 3HP, 3 months of weekly isoniazid and rifapentine; 6H, 6 months of daily isoniazid.

^a^The GDF price of rifapentine per adult 3HP regimen was 45 USD in 2018 and reduced to 15 USD for 100 countries in 2020.

^b^Includes purchasing and shipping from GDF.

## DISCUSSION

The cost of effective delivery of 3HP in Karachi, Pakistan, was lower than that of 6H, despite the higher cost of rifapentine compared to isoniazid. At the United Nations (UN) High Level Meeting on tuberculosis, countries committed to ensure that 24 million contacts of people with tuberculosis would receive preventive treatment by 2022 [20]. Concerns over cost have prevented the adoption of newer, shorter regimens. However, our study suggests that, because of higher completion, shorter regimens can cost less to deliver successfully. The non–medication-associated costs of getting contacts into care, evaluating them, and monitoring preventive treatment are higher than the cost of medicines; therefore, if few individuals complete preventive treatment, a large amount of health system effort is wasted. Moreover, our analysis highlights the economic sacrifices that patients make in accessing preventive treatment, which can exceed the cost of the medications.

Based on our estimates, assuming no additional costs associated with scale-up and no cost reductions resulting from economies of scale, the total cost of delivering tuberculosis preventive treatment to the UN target of 618 850 household contacts and people living with human immunodeficiency virus (HIV) in 2022 [21] in Pakistan would be approximately 108 million USD. Of this, the cost to the health system would be 89 million USD, which includes both existing infrastructure and new investments in medications and additional staff. The other 19 million USD represent costs to patients, which could present a barrier to delivery if programs cannot mitigate these costs.

Our analysis shows that the same economic argument used for promoting shortened regimens in wealthy countries with low tuberculosis burdens [11, 12] can be made in high-burden settings. Moreover, as in high-income settings, we found that the cost of delivering preventive treatment goes far beyond the cost of medications. In our analysis, clinical staff salaries cost approximately 18 USD per visit; in a study from Canada [12], clinician salaries cost the equivalent of 57 USD per visit in 2019 dollars. This comparison illustrates the importance of comprehensively measuring service delivery costs from a health system perspective to contextualize the contribution of medication costs. While the difference in the current price of 3HP and 6H medications is highly visible to programs and their funders, the actual difference in the costs of delivering the 2 regimens may not be as visible since program budgets may not explicitly mark nonmedication costs as being associated with preventive treatment.

Unlike previous cost comparisons of preventive treatment regimens that focused only on costs to health systems [11, 12, 22, 23], we designed our study to include the costs to patients receiving preventive treatment. Our finding that a considerable portion of total costs is incurred by patients themselves is consistent with previous studies showing that, even in health systems that offer free tuberculosis care, patients bear substantial costs for transport to health facilities and lost income [24–26]. Income loss due to clinic appointments is known to be a barrier to preventive-treatment adherence as well [6, 27]. Given that people receiving preventive treatment do not feel sick, asking them to sacrifice income to visit health facilities places a substantial burden on them that may be perceived as outweighing the benefits of preventive treatment. Shorter regimens requiring fewer follow-up visits, as well as support strategies such as transport reimbursements [28] or conditional cash transfers [29], can reduce the burden on patients to complete treatment, potentially reducing the economic cost to society as a whole of delivering preventive treatment.

Our study does not address cost-effectiveness, which we believe is both a strength and a limitation. It is a strength because we avoid making assumptions about disease progression risks or treatment efficacy, thus avoiding the uncertainty that such assumptions incur. However, by not considering outcomes other than treatment completion, we underestimate the potential benefit of better protection from tuberculosis disease given better treatment completion and thus higher effectiveness of 3HP. One cost-effectiveness analysis from the United States found that, while the cost per completed course of 3HP was only slightly lower than that of 6H, the cost-effectiveness of 3HP was much higher [22]. This result is driven by the higher effectiveness of 3HP as well as the high cost of treating tuberculosis disease should it develop. Future studies should assess the cost-effectiveness of 3HP compared with 6H in high-burden countries using completion rates, treatment costs, and costs to patients measured in these settings.

The largest source of uncertainty in our analysis was around patient costs. Self-reported costs are subject to reporting error, and the small convenience sample of the patient cost survey limits the generalizability of the results. Most of our survey respondents were guardians rather than adult patients, and we were unable to include any adults taking 6H. Therefore, the cost per visit reflects mostly responses of guardians bringing children to the clinic, and guardians may incur different costs than adults who are themselves receiving preventive treatment. In addition, we assumed uniform patient costs across the 2 regimens, although the average per-visit cost for survey respondents taking 6H was higher than for respondents taking 3HP. We were unable to explain this difference or be certain that the higher cost would have been observed when everyone was receiving 6H. We averaged costs across all respondents to avoid biasing our results in favor of 3HP. Finally, we did not ask the age and income of all family members, and therefore made simplifying assumptions about opportunity costs for people other than the survey respondent. However, we present this uncertainty by creating lower- and upper-bound estimates.

Our study is also limited by simplifying assumptions made in estimating program-level costs. We assumed that all visits for a given patient were equally costly, when, in reality, the initial visit cost more because this is when the tests involved in ruling out tuberculosis were performed. However, since the number of visits per patient was similar for the 2 regimens, this simplification is unlikely to affect the comparison between regimens. The assumption of equal costs per visit also fails to capture actual gains in efficiency experienced by the program as patient volumes increased. Due to unavailable data, we did not include costs of capital goods such as office space. However, as the preventive treatment program is built within the tuberculosis treatment services and uses the same clinic, the exclusion of capital costs should not substantially impact our results.

In conclusion, the cost of successful delivery of preventive treatment to contacts of patients with tuberculosis in Karachi, Pakistan, was lower for 3HP than for 6H due to better completion in those treated with 3HP. Medication costs represented only a fraction of the total cost of delivering preventive treatment, and patients incurred substantial costs despite free treatment. Our findings suggest that, when considering the cost of successful delivery, shorter preventive treatment regimens are more economical than 6H in high-burden settings. Policymakers and funders should take a comprehensive view of costs when considering preventive treatment regimens.

## Supplementary Data

Supplementary materials are available at *Clinical Infectious Diseases* online. Consisting of data provided by the authors to benefit the reader, the posted materials are not copyedited and are the sole responsibility of the authors, so questions or comments should be addressed to the corresponding author.

ciaa1835_suppl_Supplementary_Material

## Notes

***Financial support.*** This work was supported by the TB Modelling and Analysis Consortium (Bill and Melinda Gates Foundation; grant number OPP1084276).

***Potential conflicts of interest.*** The authors: No reported conflicts of interest. All authors have submitted the ICMJE Form for Disclosure of Potential Conflicts of Interest. Conflicts that the editors consider relevant to the content of the manuscript have been disclosed.
