# Supplementary material for: Cost of Delivering 12-Dose Isoniazid and Rifapentine Versus 6 Months of Isoniazid for Tuberculosis Infection in a High-Burden Setting
Source: Clin Infect Dis. 2020 Dec 7;73(5):e1135–41. doi: 10.1093/cid/ciaa1835 (PMC8423476; doi:10.1093/cid/ciaa1835)
Supplement: ciaa1835_suppl_Supplementary_Material [file ciaa1835_suppl_supplementary_material.docx]

**SUPPLEMENTARY MATERIAL: Detailed cost estimation methods**

**Estimating program-level costs**

*Medication-associated costs*

Medication-associated costs included the procurement and shipping cost of isoniazid and rifapentine from GDF during 2016-2018. We assumed that one full treatment course (Table S1) was used per patient initiating treatment, reflecting the practice of setting aside a full course for each patient at the time of initiation to avoid treatment interruptions in case of stock-outs. We obtained from the National Tuberculosis Program the fee structure for procuring and shipping medications from GDF and used GDF drug prices from the study period. We also conducted a sensitivity analysis using a lower price for rifapentine that became available to 100 countries in 2020.^[[1]](#footnote-1)^

**Table S1: Assumed average adult and pediatric preventive treatment regimens**

| **Age group and regimen** | **Drug** | **Doses in complete regimen** | **Dose** | **Formulation used** |
| --- | --- | --- | --- | --- |
| Adult 6H | Isoniazid | 180 | 300 mg | 300 mg uncoated tablets |
| Adult 3HP | Isoniazid | 12 | 900 mg | 300 mg uncoated tablets |
|  | Rifapentine | 12 | 900 mg | 150 mg uncoated tablets |
| Pediatric 6H | Isoniazid | 180 | 150 mg | 100 mg uncoated tablets |
| Pediatric 3HP | Isoniazid | 12 | 400 mg | 100 mg uncoated tablets |
|  | Rifapentine | 12 | 450 mg | 150 mg uncoated tablets |

*Non-medication-associated costs*

Non-medication-associated costs were collected from the records of the IHN Finance department. We identified clinical and supervisory staff involved in the preventive treatment program and the percentage of monthly effort each staff member devoted to the program. We obtained the monthly salary bracket for each identified staff member and used the midpoint of the range to estimate the monthly salary cost devoted to the program. Health workforce costs included salaries and fringe benefits for clinical and field staff time spent on contact tracing and delivering preventive treatment, as well as costs of biannual staff trainings. Supervisory costs comprised salaries of supervisory staff who oversaw the program.

The medical procedures and supplies cost category included the cost of the diagnostic tests to rule out tuberculosis disease before the initiation of preventive treatment, as well as medical supplies used during clinic visits. Diagnostic tests included those received by all patients, such as chest radiography, as well as tests received only by patients who required additional diagnostic procedures, such as GeneXpert MTB/RIF or computed tomography. We also obtained from the Finance department the costs of performing each diagnostic test, including supplies, labor, and equipment depreciation. To calculate the total costs of lab tests over the study period, we multiplied the cost of each test by the number of patients in the study cohort expected to have received them. The program did not use routine laboratory monitoring during treatment, but liver function tests could be performed if clinically indicated. However, because these tests are typically done on a very small number of patients, we did not include their costs due to the logistical challenge of identifying individual tests in the electronic medical records. For medical supplies, we obtained from the Procurement and Finance departments the monthly quantities of medical supplies used by the program and their corresponding costs.

Health information systems costs included computers, phones, and data plans used by program staff, as well as the salary of a data manager. To estimate the cost of utilities used by the preventive treatment program during clinic visits, we obtained from the Finance department the monthly utilities costs for the tuberculosis clinic as a whole. We allocated a percentage of these costs to the preventive treatment program proportional to the number of visits made for preventive treatment out of total visits to the tuberculosis clinic. As the Indus Health Network owns its facilities and no new infrastructure was required, we did not include capital costs.

We multiplied monthly non-medication costs by 17 months to estimate total non-medication costs during the study period. We then divided by the number of total preventive treatment patient visits made to obtain the per-visit cost. As non-medication costs are not sensitive to age group or treatment type, we applied the same non-medication per-visit cost estimate to both age groups and regimens.

**Estimating patient-level costs**

To estimate the expenses incurred by a patient per visit, we administered a survey to a convenience sample (N=100) of people attending preventive treatment visits at an IHN tuberculosis clinic in 2019. The sample included 50 guardians of children receiving 6H, 41 guardians of children receiving 3HP, and 9 adults receiving 3HP. Trained health workers administered the surveys after the interviewee completed a visit. At the time of this study, the program had already stopped using 6H except for children <2 years old, so we were unable to survey adult patients receiving 6H.

Survey respondents reported the total spending on medical care, round trip travel, and food or drink consumed during the visit. They also reported the amount of time spent in transit and at the hospital. We calculated the opportunity cost (lost wages) for the survey respondent by multiplying the number of hours spent by the respondent’s reported hourly wage, derived from the reported monthly wage. We asked respondents how many people accompanied them to the hospital, but not the ages or occupational status of these companions. We therefore estimated lower-bound, upper-bound, and mid-range estimates for the opportunity costs to persons who accompanied the respondent during the visit. The lower-bound estimate assumed no opportunity cost to anyone other than the respondent (e.g. a situation where the respondent was accompanied only by children). The upper-bound estimate assumed that all companions were adults with the same hourly wage as the respondent, except for the child patient if the respondent was a guardian. The mid-range estimate was the average of the high and low estimates. We then obtained the total patient-level costs incurred during the visit by summing spending on medical care, travel, food or drink, and opportunity costs. The average cost over the 100 survey respondents was used as the patient-level cost per visit. Because we did not expect patient costs to be affected by the regimen being received, we applied this cost regardless of regimen or age.

1. Global Drug Facility. July 2020 medicines catalog. Geneva: Stop TB Partnership; 2020. URL: <http://www.stoptb.org/assets/documents/gdf/drugsupply/GDFMedicinesCatalog.pdf> [Accessed 16 September, 2020] [↑](#footnote-ref-1)
